# The association of comorbidities, utilization and costs for patients identified with low back pain

**DOI:** 10.1186/1471-2474-7-72

**Published:** 2006-09-18

**Authors:** Debra P Ritzwoller, Laurie Crounse, Susan Shetterly, Dale Rublee

**Affiliations:** 1 Clinical Research Unit, Kaiser Permanente Colorado, Denver, CO, USA; 2 Pfizer Inc, U.S. Outcomes Research, NY, USA

## Abstract

**Background:**

Existing studies have examined the high prevalence of LBP along with the high treatment costs of patients with low back pain (LBP). Various factors have been shown to be correlated or predictive of chronic or episodic LBP including the characteristics of the initial episode, pain, comorbid conditions, psychosocial issues, and opiate use. This study replicates and extends earlier studies by examining the association of patient characteristics including baseline comorbidities with patterns of healthcare service use and cost.

**Methods:**

This is a retrospective analysis of measures of comorbidities, healthcare use, and cost for patients identified with LBP, stratified by the number of LBP episodes. Administrative data associated with outpatient and hospital based care for the years 1996 through 2001, were used to identify adult patients with LBP. LBP patients continuously enrolled for 12 months prior and 24 months after their initial LBP event were included in the study. A LBP episode was identified as the number of 30-day periods where a patient had one or more healthcare events with a diagnosis consistent with LBP. Chi-square and multivariate regression analyses were employed to estimate the variation in utilization and costs.

**Results:**

Of 16,567 patients enrolled, 67% were identified with only one LBP episode and 4.5% had ≥6. The prevalence of comorbidities, analgesic use, and healthcare service use, varied by the number of back pain episodes. Diabetes, rheumatoid arthritis, anxiety, psychotic illness, depression, use of opiates and NSAIDs were associated with significant incremental increases in costs (*P *< .003).

**Conclusion:**

Physical and mental health co-morbidities and measures of analgesic use were associated with chronicity, healthcare utilization and costs. Given the association of comorbidities and cost for patients with LBP, management approaches that are effective across chronic illnesses may prove to be beneficial for high cost patients identified with LBP.

## Background

Treatment of low back pain (LBP) continues to be a significant medical and financial burden. While it is one of the most common reasons for visiting a physician, observable treatment patterns remain extremely variable and expensive [1,2]. Much of the literature related to prevalence and economic burden associated with LBP has demonstrated that most patients recover within a month of the initial episode, but a small proportion go on to experience significant disability related healthcare expense [3-8]. While LBP is a common problem, consensus across the medical community with respect to prevention and treatment guidelines appears inconsistent [9]. In 1994, the Agency for Health Research and Quality (AHRQ) released clinical practice guidelines for adults with acute lower back problems. However, these guidelines are no longer viewed as guidance for current medical practice [10].

While a small percentage of patients with chronic or episodic LBP account for a large proportion of cost, significant variation exists with respect to both the definition and treatment of chronic LBP [11-15]. Various factors have been shown to be correlated or predictive of chronic LBP including the characteristics of the initial episode, pain, psychosocial issues, and occupation [11,16-18]. However, empirical evidence is mixed with respect to the association between major psychopathology, such as depression and substance abuse, and chronic LBP [16,17,19]. The relationship between pain, analgesic use and healthcare utilization related to LBP has also been explored. A study by Vogt et al demonstrated that for patients with LBP, opioid use was associated with high volume usage of LBP healthcare services and that those with psycohogenic pain were more likely to use opioids [20]. Other comorbid conditions such as respiratory disorders, heart disease and other musculoskeletal disorders have been shown to have significant association with chronic LBP [21,22]. Although physical and mental health co-morbidities are believed to play a role in the high cost cases there is little data describing their association with specific patterns of healthcare utilization that lead to higher costs [7].

In this study, we extend the current literature related to the direct medical costs associated with LBP by examining the number of back pain episodes, comorbidities, and analgesic use, and their relationship to healthcare utilization and costs for a cohort of patients diagnosed with LBP. The purpose of this study is to provide better understanding of the characteristics of patients identified with a diagnosis of LBP and their patterns of healthcare utilization and costs, who were enrolled in an integrated healthcare delivery system. We hypothesize that back pain patients with multiple co-morbidities are more likely to exhibit utilization patterns consistent with chronic LBP that in turn drive healthcare service utilization and costs.

## Methods

### Setting

We studied adult members of Kaiser Permanente Colorado (KPCO). KPCO is a group model, closed panel, non-profit HMO providing integrated healthcare services to over 410,000 (covered enrollees), approximately 15 percent of the insured population, in the Denver/Boulder, Colorado metropolitan area. KPCO has over 550 physicians in seventeen separate ambulatory medical offices spread geographically across the greater metropolitan area. Kaiser Permanente Colorado's Institutional Review Board approved this project and the associated analyses of data derived from the administrative databases.

### Study population

We identified 16,567 patients from KPCO's inpatient and outpatient based facility and professional claims, and internal outpatient primary care and specialty care databases for years 1996 through 2001. All patients were ≥18 years old at the time first index visit with a LBP diagnosis during 1997 or 1998.

### Low Back Pain diagnosis and index visit identification

A diagnosis of LBP was based on algorithm using *International Classification of Diseases, Ninth Revision *(ICD-9) codes that was developed and validated at the University of Washington and has been used in several previous studies [20,22,23]. This algorithm consists of 66 ICD9 codes that include a broad array of diagnoses deemed consistent with mechanical LBP. This algorithm excludes patients if the back pain diagnosis was secondary to major existing conditions: e.g. neoplasms, osteomyelitis, spinal abscess, pregnancy, fracture, dislocation or vehicular accident. The index visit was defined as the first contact with a healthcare provider in either an ambulatory or hospital setting, resulting in a diagnosis, either primary or secondary, of LBP that was preceded by a 12 month period of continuous enrollment with no evidence of LBP. Patients were required to be continuously enrolled for a minimum of 24 months after the index LBP event.

We created a proxy variable to capture the variation in the number of back pain episodes across the study population. This variable was defined as the number of 30-day periods where a patient had one or more healthcare events for LBP. Given the distribution of this variable (67.37% had 1, 17.43% had 2, 10.72% had 3–5, and 4.8% had 6 to 22 episodes), for analytic purposes patients were grouped into LBP episode categories of 1, 2, 3–5, and 6+. In order to better understand the distribution of the diagnoses associated with the patients' index LBP visit, we used the four diagnostic categories noted in Vogt et al (2005) [20]. These categories are (I) LBP without neurological involvement, (II) LBP with neurological involvement, (IIIa) LBP caused by congenital lumbar spinal structural disorders, (IIIb) LBP caused by acquired lumbar spinal structural disorders, (IV) and LBP due to other causes including postoperative issues. For analytic purposes, categories IIIa and IIIb were grouped together.

### Utilization measures

We captured the following measures of utilization for 12 months prior, and 24-months subsequent to the index back pain diagnosis: 1) outpatient care including primary care, specialty care, physical therapy, and mental and behavioral health; 2) all hospital based care including inpatient stays, emergency department (ED) visits, and observation stays of less than 24 hours in duration; 3) all pharmaceutical dispenses; 4) major spinal-related radiology procedures including CT and MRI.

We examined the variation in utilization patterns including outpatient primary and specialty care, mental health, physical therapy, imaging, pharmaceutical use, and hospital use by initially stratifying patients by the number of LBP episodes. Given that it is often difficult to isolate back pain specific healthcare resource use, we identified outpatient and hospital based care that was more likely to be related to back pain or musculoskeletal disorders. Outpatient care provided in orthopedics, neurology, neurosurgery, rheumatology, and physiatry departments was aggregated into an outpatient back pain specialty care category. Other specialty care included cardiology, endocrinology, gastroenterology, ophthalmology, etc. We classified hospital admissions that would fall into Major Diagnostic Category (MDC) using Diagnosis Related Groups (DRG). MDC 8 includes inpatient admissions related "*to diseases and disorders of the musculoskeletal system and connective tissue*" [24]. While this MCD in not specific to LBP, most, if not all, LBP related admissions would be captured in this diagnostic category.

### Demographic and co-morbidity measures

Gender and age at the time of the index LBP event were available from the electronic membership records. To adjust for variation in health status and prevalence of chronic conditions that could influence both healthcare service utilization and costs during the observation period, a pharmacy based risk adjustment system, called RxRisk, was used to identify comorbidities [25]. The RxRisk model, also referred to as the Chronic Disease Score, is a clinically validated algorithm that classifies patients into chronic disease categories based on prescription drug fills [26]. The RxRisk (or CDS) system is a valid and reliable predictor of future health services use and future costs. Studies using this system demonstrate that it performs as well as instruments based on International Classification of Disease, 9^th ^Revision, Clinical Modification (ICD-9-CM) inpatient and outpatient diagnoses [25,27]. Using the RxRisk algorithm, dichotomous variables were created in order to assess the contribution and association of various comorbidities to utilization and cost estimates. We estimated the prevalence of RxRisk based comorbidities for the period 12 months prior to the initial back pain index date.

We used American Hospital Formulary Service groups and the National Drug Code system to identify and classify physician prescribed pharmacy dispenses for the LBP population into categories of non-steroidal anti-inflammatory drugs (NSAIDs) and opioids. We were not able to capture over-the-counter dispenses fro NSAIDs. We estimated the prevalence of use of these products for the 12 months prior and the 12 months after the index LBP visit.

### Cost measurements

Using KPCO's cost management information system, we estimated annualized costs of care for services provided after the index back pain visit by type of utilization resource including outpatient, inpatient, hospital, pharmacy, and CT and MRI radiology procedures for the 24 months following the LBP index visit. This system allocated health care cost for all internal services provided directly by KPCO as well as claims for covered services enrollees receive from contracted providers. Internal costs are allocated by resource intensity weights (by service department and procedure) using KPCO's general ledger. Pharmacy costs are estimated using actual acquisition costs and KPCO specific pharmacy dispensing costs. All costs are reported in 1999 constant dollars. As a proxy for total annualized costs in 2005 dollars, we used data from the Medical care services component of the Consumer Price Index to inflate the 1999 cost estimates into 2005 dollars [28].

### Statistical analyses

Using chi-square tests of proportion, we compared patterns of age, gender, co-morbidities, and utilization after the initial index visit, categorized by the number of LBP episodes. Given that hospital care may be the most costly component of health service use, we employed logistic regression analyses to examine the likelihood of an inpatient admission based on age, gender and pre LBP index visit comorbidities. Because the hospital admission event may be correlated with the number of LBP episodes, we did not include this variable in the final models. Separate models predicting MDC 08 admissions were also estimated.

In order to adjust for variation in health risk that may influence cost estimates, cost is estimated as a function of age, gender, comorbidities identified prior the back pain index event, and as an extension of a study conducted by Vogt et al, we also included variables capturing a dispense for opioids or NSAIDs in the 12-month baseline period prior to the index LBP event [20]. To avoid potential confounding with the dependent variable, the number of LBP episodes were not included in the cost models. Consistent with other cost studies, the dependent variable was the total cost over the two year post index period, annualized to avoid observations with zero charges and smooth individual year-to-year random variation and to minimize the effect of outlier cost events on the overall cost distribution [29,30]. The mean and standard deviation of annualized costs, in 1999 dollars, for individuals in this cohort was $2,780 and $6,008, respectively. Given the skewness of the dependent variable, we used a weighted least squares model instead of a standard linear model [30,31]. This method involves creating a weight from the residuals from the ordinary least-squares regression to adjust for heteroskedastic error terms. This weighted least-squares regression analysis provides unbiased regression coefficients and asymptotically efficient standard errors. In addition, use of the weighted least-squares regression analysis permitted health care costs associated with the covariates in the model to remain in nominal values. The use of nominal values eliminated the need to apply a variance-stabilizing transformation to the dependent variable and subsequently retransform regression results to obtain dollar values. Parameter estimates for each variable may then be interpreted as the marginal or incremental costs associated with patient falling into that particular category.

## Results

During the observation period of January 1, 1997 through December 31, 1998, we identified a total of 16,567 patients ≥18 years, having an index visit with a diagnosis of LBP, with no LBP related visits in the previous 12 months, and who were continuously enrolled for 12 months prior to and 24 months after the index visit. Table 1 describes the distribution of age, gender, index diagnosis categories, and RxRisk comorbidity categories, stratified by the number of back pain episodes. A greater proportion of the patients were females. The mean and median age of the LBP population were 51.09 and 49.82, respectively. Age is further described within 5 year categories in Table 1. As noted earlier, the majority of patients identified (n = 11,161, 67.4%) experienced one LBP episode requiring contact with a KPCO provider during the 24 months of follow-up. Only 4.5% (n = 743) of the patients had 6 or more documented episodes. Older age was generally associated with higher numbers of LBP episodes (*P *< .001). No significant differences were found in the gender distribution of patients by number of LBP episodes.

Eighty-one percent of all index visit diagnoses fell into category I, LBP with no neurologic findings and 52 percent of all index diagnoses were coded as *Unspecific Backache *(ICD9 code 724.5). The distribution of the index visit diagnoses varied significantly by the number of observed LBP episodes (P < .001). Of those with 1 or more LBP episodes, 84% of the index visit diagnoses fell into category I, versus 57% of patients with 6 or more.

### Pharmacy based measures

We estimated prevalence for 14 comorbid conditions using the RxRisk model (Table 1) for the 12 month period prior to the LBP index visit. With the exception of cardio/peripheral vascular disease (CVD/PVD), prevalence estimates of all conditions typically increased by LBP episode category. *P*-values of < .0001 were derived from Chi-square tests for all conditions with the exception of Diabetes (*P *= .005), Thyroid disease (P = .003), and Psychosis (*P *= .073). The range of prevalence estimates for patients with 1 LBP episode relative 6 or more LBP episodes varied from 4.8% – 5.1% for Diabetes, to 17.6% – 24.4% for Hypertension.

Table 2 describes the prevalence of analgesic use in the 12 month period prior to the LBP index visit and in the 24 months after. For both NSAIDs and opioids, we found significant variation (*P *< .001) in the prevalence analgesic use, both before and after the index LBP visit and across the LBP episode categories. LBP patients having 3–5 observed LBP episodes had the largest observed increase in the prevalence of both NASIDS and opioids; 31.6% – 48.0% and 25.5% – 44.2%, respectively. While not shown in Table 2, using a 24-month post observation period we found that prevalence estimates continued to rise. Again, the highest total and largest percent increase for the 24 month period was found for patients having 3–5 observed LBP episodes (61.5% and 58.9%).

### Utilization measures

Table 3 describes percent of LBP using outpatient and hospital based care during the 24 month period after the index LBP visit, stratified by the number of LBP episodes. Significant variation (*P *< .001) across episode categories was found for all utilization measures. The prevalence of use did not always correlate with an increase in the number of LBP episodes. Use of primary care outpatient services was the highest (98%) for patients with 2 episodes, falling to 91.8% for patients with 6 or more episodes. Conversely, the percent of patients using outpatient specialty care, outpatient back pain specialty care, mental and behavioral healthcare, along with all hospital based care increased as the number of back pain episodes increased. For patients with only one LBP episode, the percent of patients with one or more hospital admissions or MDC 8 specific hospital admission, was 9.8% and 1.7%, respectively. For patients with patients with 6 or more LBP episode, the percent of patients using these services was 21.3% and 11.2%, respectively.

Results from the logistic regression model (Table 4) demonstrated that the likelihood of an inpatient admission in the two years subsequent to the index back pain event increased with age. Those aged 65–75 were twice as likely to have an admission (95% confidence interval [CI] = 1.67–3.29). For patients aged 75–85 and patients over 85, the odds increased to 4.0 and 5.46, respectively (CIs 2.82–5.65, 3.58–8.33). If the initial back pain diagnosis was in Category III or IV the likelihood also increased significantly (CI = 1.24–1.76, 1.91–3.08). Psychosis increased the likelihood by 72% (CI = 1.32–2.25), depression by 27% (CI = 1.13–1.46), asthma or COPD by 37% (CI = 1.18–1.58), diabetes by 102% (CI = 1.69–2.40), and heart disease or hypertension by 72% (CI = 1.53–1.94). The use of opiates or NSAIDs prior the index back pain event increased the likelihood of an inpatient admission by 41% (CI = 1.25–1.59) and 19% (CI = 1.06–1.33), respectively. The effects of older age, diagnoses category III and IV, and NSAID use were even more pronounced for the outcome of an MDC 8 admission. However, the effects of the comorbidities noted above, were not significant. While not included in the final models shown in Table 4, the likelihood of any inpatient admission and an inpatient admission for MDC 8, increased significantly by the number of observed LBP episodes months in the 24-month post index period. Relative to patients with only one LBP episode, those with 6 or more were 90% more likely (P < .0001) to have any inpatient admission, and almost 6 times more likely to have an admission for MDC 8 (P < .0001).

### Cost analyses

**Figure 1:**
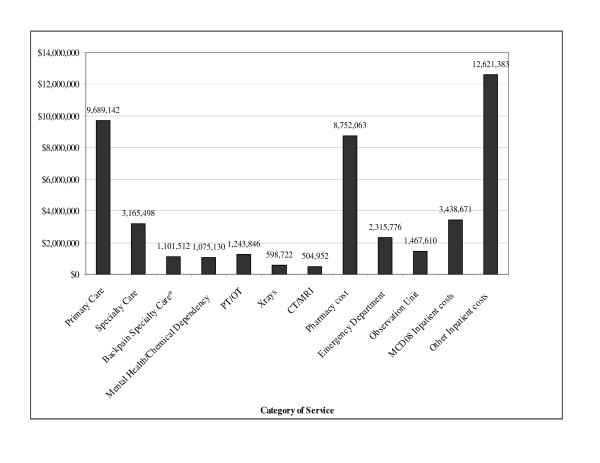
Average annual total costs for $16,567 LBP patients by category of service.

Costs were initially estimated by category of service: Hospital, outpatient, radiology, and pharmacy. Total annualized average costs for the 24 months post LBP index visit, estimated in 1999 constant dollars, were $45,974,305. Using the Medical Care component of the CPI-U as a proxy for changes in medical care service costs, this number almost doubles to $70,934,545 in 2005 dollars. The distribution of these costs by service use category, are demonstrated in Figure 1. Primary care utilization was the largest component of outpatient costs. Back pain specialty care, mental health/chemical dependency, PT/OT, and radiology cost were each all estimated to be over $1 million dollars annually. Not unexpectedly, inpatient care was the largest overall cost component, with an annualized cost of over $16 million. Stratifying by the number of back pain episodes, this translates to a total average annual per patient cost of $2,410 ($3,718 in 2005 dollars) for patients with 1 LBP episode, $3,114 ($4,805 in 2005 dollars) for patients with 2 LBP episodes, $3,807($5,874 in 2005 dollars) for patients with 3–5 LBP episodes, and $4,464 ($6,888 in 2005 dollars) for patients with 6 or more LBP episodes.

Table 5 describes the results from the annualized total cost weighted least squares regression model. As noted by the R^2^, the model explained 31.63 percent of the variation in annualized total costs. This level of explanatory power is not inconsistent with other predictive cost models [25,32,33]. Total annualized costs increased with female gender, older age, and comorbidities and analgesic dispenses identified in the 12 month period prior to the index visit. An estimated incremental increase of $2,001 was associated with the initial index diagnoses falling into Category IV, or other non-specific conditions. At $2,799, diabetes had the highest incremental cost estimate compared to other comorbidities included in the model. While rheumatoid arthritis was not significant in the inpatient MDC 08 logistic models, the marginal cost of this comorbidity was large and statistically significant ($1,625, p-value < .0001). Mental illness as proxied by the RxRisk categories identifying anxiety, psychotic illness, and depression in the period prior to the first back are also associated with significant marginal costs. The use of opiates and NSAIDs was also significant at (p-value <.0001) $912 and $524, respectively.

## Discussion

Our findings from this study are consistent with the results found in other studies that examine treatment patterns and costs associated with LBP. Consistent with seminal study of Engel et al [7] that examined the predictors of high healthcare costs for patients diagnosed with LBP, we found that a small proportion of patients with multiple LBP episodes had higher costs. Also consistent with our results, in a study examining the relationship of comorbidities and LBP episodes, Nordin et al (2002) found that the presence of any comorbidity at the index visit was associated with significantly longer duration of LBP related work disability [22]. We also observed an association of depression and psychopathology with an increased number of LBP episodes and costs that was demonstrated in three other studies [7,34,35].

The results show a relatively high prevalence of opioid use in the population with multiple LBP episodes. While the prevalence estimates of NSAID and opioids use that we noted here are not inconsistent with those presented by Vogt et al [20], evidence suggests that these agents may pose a risk to patients [36-38].

Inpatient admissions for falling into the category of MDC 8 made up 23% of all inpatient admissions in the 24-month post-index LPB event. It is not surprising that many of the disease indicators which were associated with any hospitalization were not significantly associated with the MDC 8 admissions. The stronger association for dispenses for NSAID and opioids the baseline period (prior to the index LBP event) in the MDC 8 models likely reflect elements of severity contributing to the MDC 8 admissions.

This study has several limitations. It relies on administrative data rather than on patient report of the initial and subsequent LBP episodes. It also lacks self-report data related to pain and disability. While we limited the sample to those with no documented contact with a healthcare provider associated with LBP, we cannot assume that the index visit is the first episode of LBP for the population that we studied. In addition, our measure of the number of LBP episodes, number of 30 day periods with one or more LBP events, may not be consistent with episode definitions in other studies and we cannot be sure they represent separate unique episodes [11,12,16]. This variable may also be capturing patient visits for multiple acute episodes, particularly if they occur at the large time intervals. A value of 2 could reflect two visits a week about one on each site of the window border or two visits in a year apart. In this analysis, LBP episodes are likely to capture elements of both chronicity and severity. What these elements may imply about a patient's low back pain condition is not examined in this manuscript. Our capture of NSAIDs was limited to physician prescribed dispenses. We did not observe over-the-counter purchases of NSAIDs. We only examined factors associated with chronicity and cost, rather than estimating a prediction model of patients who would go on to have multiple LBP episodes related high costs. We limited the cost analyses to the direct medical costs borne by the payer or insurer. These cost estimates did not include complementary and alternative medical treatments including chiropractry, acupuncture, or massage therapy, which may now be a covered benefit for some LBP patients [39]. We did not examine the indirect costs to the patient associated with pain, disability, loss of work and leisure activities, etc. We also did not examine the indirect costs to society associated with absentisem and loss of productivity.

## Conclusion

To our knowledge, this is the largest study to examine the distribution of comorbidities and analgesic use, the number of LBP episodes and their association with healthcare utilization and costs. We demonstrated that most measures of utilization and annual total costs increased with age, comorbidities, use of analgesics, and the number of LBP episode months.

We estimated that in 2005 dollars the annual direct medical costs for 16,567 patients who present with LBP was $70,934,545, or on average $357 per member, per month. These patients are very expensive and they are very complex with respect to the prevalence and distribution of other comorbidities. Our utilization estimates demonstrated that those with most LBP episodes were the lowest users of primary care, but the highest users of all forms of specialty care. The findings from this study, reinforce the suggestions by Carey and Freburger (2005), and Nordin et (2002) that special attention to high utilizers, and those with other chronic conditions may improve the outcomes for these patients, and possibly reduce both the short and long term costs associated with this condition [40,22]. Given the general lack of consensus on guidelines for the management of back pain, and the comorbidity patterns that we found, it would make sense to rely on management approaches that seem effective across chronic illnesses. To our knowledge, the Chronic Care Model [41] has received the greatest attention and research among general evidence-based approaches to disease management. The Chronic Care Model also seems particularly applicable with respect to LBP due to its emphasis on self-management and self-management support [42].

## Competing interests

Debra P. Ritzwoller, Laurie Crounse, and Susan Shetterly have no competing interest. Dale Rublee is an employee of, and holds stock in, Pfizer Inc.

## Authors' contributions

DPR was responsible for the design and coordination of the analyses. DR provided feedback and suggestions associated with the study design, interim analyses, and various drafts of the manuscript. DPR performed the inpatient and cost analyses. LC completed data extraction, descriptive analyses and participated in the study design. SS completed selected analyses that assisted in paper direction. All authors participated in writing the manuscript and have read and approved the final manuscript.

## Pre-publication history

The pre-publication history for this paper can be accessed here:



## Figures and Tables

**Table 1:** Distribution of Age, Gender Index LBP Diagnostic Category, and RxRisk Comorbidty Category by LBP Episodes for 16,567 Patients Identified with LPB

	**Number of LBP Episodes**					
	**1 ***(11,161)*	**2 ***(2887)*	**3–5 ***(1776)*	**6+ ***(743)*	**All ***(16,567)*	**Chi-Sq **P-value
**(N)**	**%**	**%**	**%**	**%**	**%**	
*Age at First Back Pain*						
18–24 *(752)*	5.2	4.1	2.9	1.1	4.5	
25–34 *(1760)*	11.3	10.1	8.8	7.3	10.6	
35–44 *(3668)*	23.4	21.0	17.8	18.6	22.2	
45–54 *(4116)*	24.6	24.0	27.0	27.1	24.8	
55–64 *(2759)*	16.3	17.0	18.2	17.4	16.7	
65–74 *(2221)*	12.2	15.2	16.0	18.7	13.4	
75–84 *(1051)*	5.8	7.2	7.6	8.8	6.3	
85+ *(240)*	1.4	1.5	2.0	1.2	1.5	<.0001
						
Male *(7679)*	46.9	45.6	45.4	43.3	46.4	0.087
						
*Index Diagnosis Category*						
I (*13,416*)	84.0	81.2	72.0	56.7	81.0	
II (*1,592*)	8.6	9.6	14.5	12.7	9.6	
III: (*1,114*)	5.7	7.1	10.6	26.2	6.9	
IV: (*445*)	2.7	2.2	2.9	4.6	2.5	<.0001
						
Prevalence Of RxRisk Comorbidity*						
Anxiety (*2026*)	10.8	14.9	15.8	15.5	12.2	<.0001
Asthma/COPD (*1978*)	11.2	13.1	14.1	14.4	12.0	<.0001
Cardiac Disease (*1134*)	6.4	7.3	8.3	9.0	6.8	0.0005
CVD/PVD (*495*)	2.8	3.4	3.2	3.9	3.0	0.10
Depression (*2227*)	12.3	14.5	16.6	18.6	13.4	<.0001
Diabetes (*854*)	4.8	5.8	6.5	5.1	5.2	0.004
GI Disease (*2100*)	12.5	13.6	16.2	18.6	12.7	<.0001
HD/Hyperten (*2327*)	13.1	15.5	16.6	16.7	14.0	<.0001
Hyperlipidemia (*1035*)	5.7	6.8	7.1	10.0	6.2	<.0001
Hypertension (*3212*)	17.6	21.0	26.1	24.4	19.4	<.0001
Inflammation (*2598*)	14.0	18.3	20.6	18.6	15.6	<.0001
Psychosis (*398*)	2.2	2.6	3.0	3.1	2.4	0.073
Rheum Arthritis (*732*)	3.9	5.4	5.6	5.7	4.4	<.0001
Thyroid Disease (*1301*)	7.4	8.3	8.9	10.2	7.9	0.003

*Comorbidity abbreviations:

COPD = Chronic Obstructive Lung Disease

GI = Gastrointestial

HD/Hyperten = Heart Disease/Hypertension

Rheum= Rheumatiod

**Table 2:** Prevalence of Analgesic Use – 12 months Prior and 24 Months After the LBP Index Visit

		**% Any NSAID Pharmacy Dispense**		**% Any Opioid Pharmacy Dispense**	
		**Pre %**	**Post %**	**Pre %**	**Post %**

*Full Sample LBP Episodes*	*(16,567)*	23.8	30.8	20.0	28.5
1	*(11161)*	21.4	25.0	18.0	24.0
2	*(2887)*	26.8	39.3	21.3	33.1
3–5	*(1776)*	31.6	48.0	25.5	44.2
6+	*(743)*	29.7	44.0	26.6	42.3

**Table 3:** Service Use by Number of LBP Episodes in the 24 Months Post LBP Index Visit

	**1 ***(11,161) *(% of Patients)	**2 ***(2,887) *(% of Patients)	**3–5 ***(1,776) *(% of Patients)	**6+ ***(743) *(% of Patients)	**Total ***(16,567) *(% of Patients)	***Chi-Sq ***P-Value
Category *						
Primary care	93.3	98.0	97.2	91.8	94.5	0.004
Specialty Care	41.2	47.6	57.8	59.4	44.9	<.0001
BP Spec Care*	12.9	17.6	27.4	35.3	16.3	<.0001
PT/OT	17.9	31.0	45.3	40.1	24.1	<.0001
Mental Health	9.3	12.0	10.9	13.7	10.1	<.0001
CT/MRI	4.6	14.0	34.0	37.3	10.9	<.0001
Spine X-ray	6.3	17.4	30.5	31.9	12.0	<.0001
ED/Observation	27.5	36.0	37.6	35.7	30.4	<.0001
Hospital Admit	9.8	12.1	14.2	21.3	11.2	<.0001
Hospital Admit for MDC 8	1.7	2.4	5.2	11.2	2.6	<.0001

*Back Pain Specialty includes orthopedics, neurology, neurosurgery, rheumatology, and physiatry outpatient departments. PT/OT refers to Physical Therapy or Occupational Therapy. ED/Observation refers hospital based care, < 24 hrs in duration in the Emergency Department or Observation Unit.

**Table 4:** Inpatient Logistic Regression Model

	All Inpatient Admits Number of Events = 1,855		MDC 08 Inpatient Admits Number of Events = 433	
**Variable Label**	**Odds Ratio**	**Confidence Intervals**	**Odds Ratio**	**Confidence Intervals**

Male (Female ref)	1.09	0.98–1.2	1.22	0.99–1.49
Age 18–24(ref)	-		-	
Age 25 – 34	0.87	0.60–1.28	0.47	0.14–1.55
Age 35 – 44	0.81	0.60–1.26	1.45	0.57–3.71
Age 45 – 54	1.16	0.83–1.62	2.09	0.83–5.23
Age 55 – 64	1.56	1.12–2.18	3.45	1.38–8.63
Age 65 – 74	2.35	1.67–3.29	5.11	2.04–12.76
Age 75 – 85	4.00	2.82–5.65	11.26	4.50–28.21
Age > 85	5.46	3.58–8.33	12.57	4.67–34.16
Dx Category II (I=ref)	1.00	0.84–1.19	1.64	1.22–2.20
Dx Category III	1.48	1.24–1.76	1.74	1.27–2.39
Dx Category IV	2.43	1.91–3.08	5.64	4.09–7.78
Anxiety	1.14	0.99–1.32	1.17	0.89–1.53
Psychosis	1.72	1.32–2.25	1.38	0.81–2.35
Depression	1.27	1.13–1.46	0.96	0.74–1.27
Asthma/COPD	1.37	1.18–1.58	1.22	0.92–1.60
Diabetes	2.02	1.69–2.40	1.14	0.80–1.63
GI Disorder	1.12	0.97–1.28	1.10	0.86–1.43
HD/Hypertension	1.72	1.53–1.94	1.19	0.95–1.49
Rheumatoid Arthritis	1.36	1.10–1.68	1.11	0.74–1.65
Opiate dispense	1.41	1.25–1.59	1.27	1.02–1.59
NSAID dispense	1.19	1.06–1.33	1.76	1.43–2.17

Comorbidity abbreviations: COPD = Chronic Obstructive Lung Disease, GI = Gastrointestinal, HD/Hyperten=Heart Disease/Hypertension

**Table 5:** Annualized Total Cost Weight Least Squares Regression Model N= 16,547 Adjusted R^2 ^= 0.3163

**Variable Label**	**Parameter Estimate**	**Standard Error**	**P-Value**
Intercept	-1241.14	119.66	<.0001
Male	-294.21	50.95	<.0001
Age 25 – 34	-164.83	137.67	0.23
Age 35 – 44	-109.54	126.44	0.81
Age 45 – 54	30.81	132.77	0.02
Age 55 – 64	302.04	132.77	<.0001
Age 65 – 74	1203.64	138.42	<.0001
Age 75 – 85	1936.07	156.66	<.0001
Age > 85	1800.55	238.81	<.0001
Diagnosis Category II	120.44	83.86	0.15
Diagnosis Category III	156.54	99.26	0.11
Diagnosis Category IV	2001.02	152.28	<.0001
Anxiety	827.58	79.58	<.0001
Psychotic Illness	1571.03	162.47	<.0001
Depression	1430.44	76.67	<.0001
Asthma/COPD	660.79	79.00	<.0001
Diabetes	2937.67	114.06	<.0001
GI Disorder	1013.77	76.72	<.0001
HD/Hypertension	1339.12	63.83	<.0001
Rheumatoid Arthritis	1625.42	124.42	<.0001
Thyroid Disorder	250.62	94.15	0.007
Opiate dispense	911.93	64.47	<.0001
NSAID dispense	524.73	59.74	<.0001

*Reference Categories include in the intercept parameter estimate were Female, Age 18–25, and Diagnosis Category I.
